# Two new species of Hygrocybe subg. Hygrocybe (*Hygrophoraceae*, *Agaricales*) from southeast China

**DOI:** 10.3897/mycokeys.142.209944

**Published:** 2026-10-21

**Authors:** Dong-Yang Xu, Xiao-Chen Hu, Xie Wu, Jia Liu, Jian-Ping Zhou, Ming-Hui Chen, Zhi-Jun Zhai, Jun-En Huang, Chen-Wei Liu, Hai-Yan Song, Dian-Ming Hu

**Affiliations:** 1 Bioengineering and Technological Research Centre for Edible and Medicinal Fungi, Jiangxi Agricultural University, Nanchang 330045, China Bioengineering and Technological Research Centre for Edible and Medicinal Fungi, Jiangxi Agricultural University Nanchang China; 2 Nanchang Key Laboratory of Edible and Medicinal Fungi, Jiangxi Agricultural University, Nanchang 330045, China Nanchang Key Laboratory of Edible and Medicinal Fungi, Jiangxi Agricultural University Nanchang China; 3 Jiangxi Provincial Key Laboratory of Excavation and Utilization of Agricultural Microorganisms, Nanchang 330045, China Jiangxi Provincial Key Laboratory of Excavation and Utilization of Agricultural Microorganisms Nanchang China; 4 Department of Biotechnology and Food Engineering, Guangdong Technion-Israel Institute of Technology, Shantou, 515063, China Department of Biotechnology and Food Engineering, Guangdong Technion-Israel Institute of Technology Shantou China; 5 Nanjing Institute of Environmental Sciences, Ministry of Ecology and Environment, Nanjing 210042, China Nanjing Institute of Environmental Sciences, Ministry of Ecology and Environment Nanjing China

**Keywords:** Morphology, new taxa, phylogeny analysis, taxonomy, waxcap

## Abstract

The genus *Hygrocybe* is a species-rich group of macrofungi widely distributed across the world; however, species within this genus show high morphological variability and significant regional differences, and taxonomic revisions are ongoing. In this study, based on specimens from Anhui and Jiangxi provinces, China, we describe two previously unreported species, *Hygrocybe zhibaiensis* and *H. longchuanensis*, using morphological studies and phylogenetic analyses based on internal transcribed spacer (ITS) and large subunit ribosomal (LSU) sequences. *Hygrocybe zhibaiensis* is characterized by viscid basidiomata, a greenish pileus, a stipe that is bright yellow at the apex and gradually fades to white downwards, and basidiomata that turn black upon bruising or with age; *H. longchuanensis* is characterized by a bright red pileus at the center with an orange-yellow margin, a viscid surface, and an orange-yellow to yellow stipe, Basidiospores ellipsoid to subglobose, no clamp connections. This discovery enriches the diversity of *Hygrocybe* in southeastern China and contributes to the taxonomic research of this genus.

## Introduction

*Hygrocybe* (Fr.) P. Kumm. (*Basidiomycota*, *Agaricomycetes*, *Agaricales*, *Hygrophoraceae*) was established in 1871, with *Hygrocybe conica* (Schaeff.) P. Kumm. (1871) as the type species. The genus *Hygrocybe* is widely distributed worldwide, with records ranging from tropical to frigid regions (Boertmann 2010). Fungal Names currently records 388 current species names for *Hygrocybe* (https://nmdc.cn/fungalnames/; accessed on 24 Sep. 2026) and Index Fungorum have 771 results for *Hygrocybe* (https://www.indexfungorum.org; accessed on 24 Sep. 2026).

*Hygrocybe* species typically grow in unimproved grasslands and are therefore considered ecological indicators (Griffith et al. 2002; Wood et al. 2017). Historically, different views have been proposed regarding the nutritional mode of *Hygrocybe*. Species of this genus are commonly found in grasslands and were thus considered saprotrophic fungi. However, Griffith et al. (2002) identified a distinctive C and N isotope pattern using isotope ratio mass spectrometry, suggesting that *Hygrocybe* is not saprotrophic. Recent studies suggest that it is neither saprotrophic nor a typical ectomycorrhizal fungus, but rather a biotrophic endophyte, possibly forming mycorrhizal associations with plants (Seitzman et al. 2011; Halbwachs et al. 2013, 2018).

According to Lodge et al. (2014), *Hygrocybe* has two subgenera: subgen. *Hygrocybe* (Fr.) P. Kumm., 1871 and subgen. *Pseudohygrocybe* Bon, 1976. Subgen. *Hygrocybe* is characterized by: pileus is brightly colored, rarely colorless, mostly red, orange, yellow, purple, or green, becoming black with age or when bruised. Lamellae are free or narrowly attached, rarely broadly attached. Lamellar trama is parallel, usually with tapered ends and exceeding 140 μm (some >1000 μm) in length, with or without transverse septa. Clamp connections are usually present in the context and hymenium, unless the spores are ornamented with spines or the basidia are bisporic. (Lodge et al. 2014).

In this study, several *Hygrocybe* specimens from Anhui and Jiangxi provinces were investigated. Through morphological and phylogenetic analyses, the specimens were determined to represent two new species of subgen. *Hygrocybe*: *Hygrocybe zhibaiensis* and *Hygrocybe longchuanensis*.

## Materials and methods

### Specimen collection

The studied macrofungi specimens were collected in Anhui and Jiangxi Provinces, China. During collection, habitat photographs were taken, the growth environment, the elevation, latitude and longitude date at the collection site were recorded. Specimens were excavated with a shovel, placed in plastic boxes after recording, then the samples were dried at 46–48 °C for 24 hours until completely dry (Liao et al. 2018; Wang et al. 2020; Hu et al. 2022; Rathnayaka et al. 2025; Li and Yang 2026).

### Macroscopic and microscopic studies

Macroscopic features of the samples were recorded from fresh samples on field following Liao et al. (2018). Color descriptions follow Kornerup and Wanscher (1981). Microscopic features of the specimens were recorded after rehydration in a 5% potassium hydroxide (KOH) solution and stained with a 1% ammoniacal Congo red solution. Freehand sections were made using a Nikon SMZ1270 stereomicroscope in accordance with the standardized method described in previous studies (Wang et al. 2015; Guo et al. 2025).

The microscopic features were observed using a Nikon Y-TV55 compound microscope. The dimensions of basidiospores are provided as (a–)b–c(–d) × (e–)f–g(–h), with c representing the average size, and the range b–d representing a minimum of 90% of the measured values (5^th^–95^th^ percentile). Extreme values (a and e), if present (a<5^th^ percentile, d>95^th^ percentile), are enclosed in parentheses. Q denotes the length/width ratio for spores, while Q_m_ refers to the average Q value for basidiospores ± sample standard deviation (Wang et al. 2019, 2020). Other measurements follow a similar format as presented by Zhou et al. (2023). The dried specimens were deposited in the Fungal Herbarium of Jiangxi Agricultural University (HFJAU).

### DNA extraction, PCR amplification and sequencing

DNA was extracted from the dried specimens with the CTAB method (Doyle and Doyle 1987; Huang et al. 2000). The internal transcribed spacer (ITS) region, was amplified using the primer pair ITS1/ITS4 and large subunit ribosomal (LSU) region was amplified with the primer pair LROR/LR5 (White et al. 1990; Huang et al. 2000). The PCR reaction in this study was 25 μL mixture consisted of 1 μL DNA template, 1 μL each primer (X μM), 12.5 μL Taq polymerase, 9.5 µL ddH_2_O (Zhou et al. 2023), PCR amplification was performed using the following thermocycling conditions: pre-denaturation at 94 °C for 3 min and denaturation at 94 °C for 30s, annealing at 55 °C for 50s, extend at 72 °C for 1 min, the total number of cycles from step denaturation to extension is 39 cycles at 72 °C, and a final extension of 10 min at 72 °C, finally, keep at 12 °C (Zhou et al. 2023). PCR products were detected in 1% agarose gels, and then sent to TSINGKE Biotechnology for both directions sequencing (Zhou et al. 2023). The primers used for sequencing were the same as those for PCR amplification.

### Phylogenetic analyses

To identify taxa closely related to our new species, we performed BLASTn searches of the obtained raw sequences against GenBank. All sequences used in this analysis, including reference sequences from (Lodge et al. 2014; Wang et al. 2020; Guo et al. 2025; Li and Yang 2026), were retrieved from GenBank (Table 1). Sequence alignment was performed using MAFFT version 7 online (https://maft.cbrc.jp/alignment/server/) (Katoh and Standley 2013) and subsequently visualized and edited with BIOEDIT v7.0.9 (Hall 1999).

**Table 1.** Details of sequences used in the phylogenetic analyses. The new generated sequences in this study are shown in red.

**Taxon name**	**Voucher numbers**	**Country of origin**	**GenBank numbers**	
			**ITS**	**LSU**
*Hygrocybe acutoconica*	520621MF381	unkonwn	PP454979	–
H. acutoconica var. microspora	TENN 010798	USA	NR_119893	–
*H. acutoconica*	G0155	Norway	–	MK278174
*H. acutoconica*	PRM 954266	Czech Republic	–	PP102486
*H. acutoconica*	PRM 954266	Czech Republic	–	OR602414
H. acutoconica var. microspora	OMDL iNat # 176367795	USA	OR824663	–
H. acutoconica var. microspora	OMDL iNat # 173306097	USA	OR785940	–
H. acutoconica var. pallidocarnea	SIENA-5495	Italy	PP965547	PP965550
H. acutoconica var. pallidocarnea	AH39432	Spain	PP965551	PP965546
H. aff. astatogala	MEL 2382984	Australia	KP012839	–
H. aff. citrinovirens	DJL05TN10 (TENN)	USA	KF291090	KF291091
H. aff. conica	AFTOL-ID 729	unknownnn	AY854074	–
H. aff. conica	TFB14689	USA	–	MN058728
H. aff. hypohaemacta	iNat63858864	USA	OP028520	–
H. aff. occidentalis	JS591	Brazil	PP883766	–
H. aff. prieta	DJL-BZ-65	Belize	KF291168	KF291169
*H. appalachianensis*	UNCA00420	USA	PZ011164	–
*H. appalachianensis*	GRSM77069	USA	FJ596915	–
*H. appalachianensis*	AK-111	USA	–	KF381547
*H. astatogala*	OTA:75752	New Zealand	PV454058	–
*H. astatogala*	KUBOT-KRMK-2020-104	India	MW491762	MW491761
H. cf. conica	Mushroom Observer 326626	USA	MK305940	–
H. cf. conica	CMFR NC-89	USA	KF291176	–
H. cf. conica	OTU_11255	Canada	–	KY353779
H. cf. flavescens	Mushroom Observer # 305807	USA	MN089496	–
H. cf. flavescens	DJL04NC4 (CFMR NC-255)	USA	–	KF291121
H. cf. nigrescens	MushroomObserver.org/306102	USA	MH128161	–
*H. chloochlora*	SAC-PR-9899 (CFMR PR-5350)	Puerto Rico	KF381522	–
*H. chlorophana*	CORS: FNG/NS/23013130	France	PZ340379	–
*H. chlorophana*	CORS: FNG/MH/24103003	France	PZ340241	–
*H. chlorophana*	OMDL iNat # 310065288	USA	PX281527	–
*H. chlorophana*	iNat59221379	USA	ON129275	–
*H. chlorophana*	FA 1806	France	OR419881	–
*H. chlorophana*	1.3.192-3	Switzerland	OP538827	–
*H. chlorophana*	2.2.978	Switzerland	OP538819	–
*H. chlorophana*	1.2.86	Switzerland	OP538816	–
*H. chlorophana*	2.2.520-1	Switzerland	OP538808	–
*H. chlorophana*	PRM 952546	Czech Republic	–	OR602408
*H. chlorophana*	PRM 952546	Czech Republic	–	PP102479
*H. chlorophana*	G0532	Sweden	–	MK278164
*H. citrinovirens*	1.3.140	Switzerland	OP538820	–
*H. citrinovirens*	1.2.126	Switzerland	OP538807	–
*H. citrinovirens*	G0454	Germany	–	MK278165
*H. coccinea*	SNMH713	Slovakia	PP762522	PP762510
*H. coccinea*	HMJAU59792	China	PX411682	–
*H. coccinea*	SNMH668	Greece	–	PP762509
*H. conica*	HMJAU66510	China	OQ927078	–
*H. conica*	HMJAU61878	China	ON045774	–
*H. conica*	F043	Czech Republic	–	PP102546
H. conica var. conica	Rotheroe-MR3	UK	KF545860	–
H. conica var. conicoides	RBG Kew K(M)64131	unknown	EU784300	–
H. conica var. conicoides	13057	Italy	JF908055	–
H. conica var. conicoides	H76	Tunisia	PP533006	–
H. conica var. conicoides	QHU20235	China	OM970939	OM942720
H. conica var. conicoides	122	USA	MK627507	–
H. conica var. conicoides	G0299	Hungary	–	MK278167
*H. cortinarioides*	HKAS:141677	China	NR_202959	PX910605
*H. cortinarioides*	HKAS83725	China	PX915265	PX910611
*H. debilipes*	GDGM59314	China	MW001783	MW007877
*H. debilipes*	GDGM57013	China	MW001782	MW007875
*H. debilipes*	GDGM59133	China	MW001784	MW007876
*H. flavescens*	MICH340319	USA	OM985890	–
*H. flavescens*	SAT-04-051-01	USA	MZ054336	–
*H. flavescens*	MushroomObserver.org/389226	USA	MN604691	–
*H. flavescens x H. chlorophana*	TENN61898	USA	JF313719	–
*H. glutinipes*	DJH24-13	UK	PQ737152	–
*H. glutinipes*	KN_003	Switzerland	OP538719	–
*H. glutinipes*	RBG Kew K(M)128171	unknown	EU784312	–
*H. glutinipes*	G0153	Norway	–	MK278169
*H. griseonigricans*	GDGM73527	China	MW001790	MW007883
*H. griseonigricans*	GDGM81385	China	MW001791	MW007882
*H. griseonigricans*	GDGM81394	China	MW001792	MW007884
*H. hypohaemacta*	CFMR BZ-1903	Belize	EU435150	–
*H. hypohaemacta*	KUM61079 (CFMR)	Malaysia	–	KF381551
*H. intermedia*	SNMH686	Slovakia	PP762525	PP762513
*H. intermedia*	SNMH687	Slovakia	PP762524	PP762512
*H. konradii*	SM670(B436b)	Malta	OR567847	–
*H. konradii*	SM670(B436a)	Malta	OR567846	–
H. konradii var. konradii	Boertmann 2004/6	Denmark	KF306329	KF306330
H. konradii var. konradii	4370	Italy	JF908058	–
*H. longchuanensis*	HFJAU15442	China	PZ744122	PZ741692
*H. longchuanensis*	HFJAU15445	China	PZ744118	–
*H. longchuanensis*	HFJAU15443	China	–	PZ741693
*H. longchuanensis*	HFJAU15444	China	–	PZ741694
*H. nanfengmianensis*	HFJAU15000	China	–	PV798754
*H. nanfengmianensis*	HFJAU15001	China	–	PV798755
H. nigrescens var. brevispora	TWH1029	Brazil	PP830807	–
H. nigrescens var. brevispora	SAC5	Puerto Rico	KF291130	KF291131
H. nigrescens var. brevispora	ECO-TA-HO 7868	Mexico	MF156250	–
*H. noninquinans*	CFMR-PR-6372	Puerto Rico	–	KF291129
*H. persistens*	KN_1149	Switzerland	OR194908	–
*H. persistens*	MEL:2416713	Australia	–	OL771765
H. persistens var. persistens	H1	Hungary	FM208852	–
H. persistens var. persistens	H23	Hungary	FM208872	–
*H. pseudoacutoconica*	GDGM 57214	China	NR_166248	MH885517
*H. pseudoconica*	5159	Italy	JF908060	–
*H. pseudoconica*	G0500	Germany	–	MK278168
*H. pseudoconica*	LE 3601	Russia	–	KF291190
*H. rubroconica*	GDGM45213	China	MW001786	MW007881
*H. rubroconica*	GDGM44706	China	MW001788	MW007878
*H. rubroconica*	GDGM45209	China	MW001787	MW007879
*H. rubroconica*	GDGM45214	China	MW001789	MW007880
*H. singeri*	HMJAU61918	China	ON045828	ON046336
*H. singeri*	FDS-CA-01457	USA	PQ144465	–
*H. singeri*	SFC20210902-20	South Korea	ON059527	–
*H. singeri*	G1994	USA	–	MK278179
*H. sinorubra*	HKAS:142439	China	NR_202958	PX910599
*H. sinorubra*	HKAS150872	China	PX915258	PX910604
*H. spadicea*	25-Oct-2006 (ABS)	UK	KF291201	–
*H. spadicea*	26-Oct-2006 (ABS)	UK	KF291199	–
*H. spadicea*	SNMH695	Slovakia	PP762531	PP762519
*H. spadicea*	DAOM171030	unknown	–	AF261451
H. spadicea var. spadicea	H32	Hungary	FM208879	–
*H. virescens*	OMDL iNat # 193004329	USA	PX105562	–
*H. virescens*	OMDL iNat # 192499992	USA	PQ615624	–
*H. zhibaiensis*	HFJAU15439	China	PZ744119	PZ741690
*H. zhibaiensis*	HFJAU15440	China	PZ744120	PZ741691
*H. zhibaiensis*	HFJAU15441	China	PZ744121	–

Phylogenetic analyses were referred to Dissanayake et al. (2020) and conducted using maximum likelihood (ML) and Bayesian posterior probability (BYPP) algorithms on the CIPRES Science Gateway portal (https://www.phylo.org/). Maximum likelihood analyses were performed using RAxML- HPC v.8, with rapid bootstrap analyses, followed by 1000 bootstrap replicates using the GTRGAMMA substitution model. The Bayesian analyses were performed using MrBayes 3.2.7a, and the best nucleotide substitution model for each data partition was evaluated with MrModeltest 2.2 (Nylander 2004). The Markov Chain Monte Carlo (MCMC) sampling approach was used to calculate posterior probabilities (BYPP) (Rannala and Yang 1996). Six simultaneous Markov chains were run for 2 million generations, and trees were sampled every 1000^th^ generation. The average standard deviation of split frequencies fell below 0.01, indicating convergence among the independent runs.

## Results

### Phylogenetic analyses

**Figure 1.**
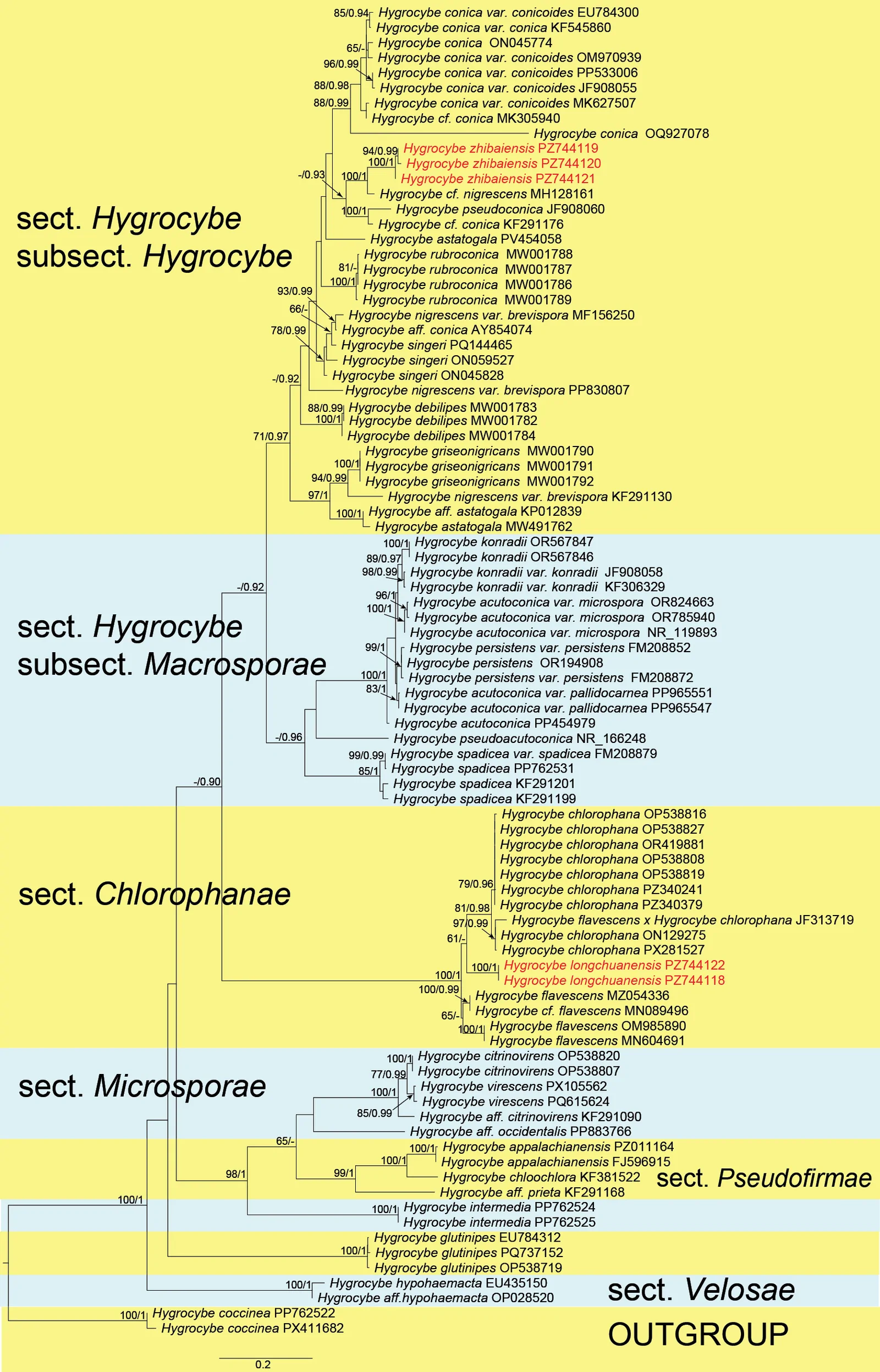
Phylogram generated from maximum likelihood (ML) analysis based on the combined ITS sequence data. Bootstrap support values for maximum likelihood (ML) equal to or greater than 60% and Bayesian posterior probabilities (BYPP) equal to or greater than 0.90 are defined above the nodes as ML/BYPP. New species are indicated in red. The tree is rooted to *Hygrocybe coccinea* (Schaeff.) P. Kumm. (1871) (outgroup PP762522, PX411682).

**Figure 2.**
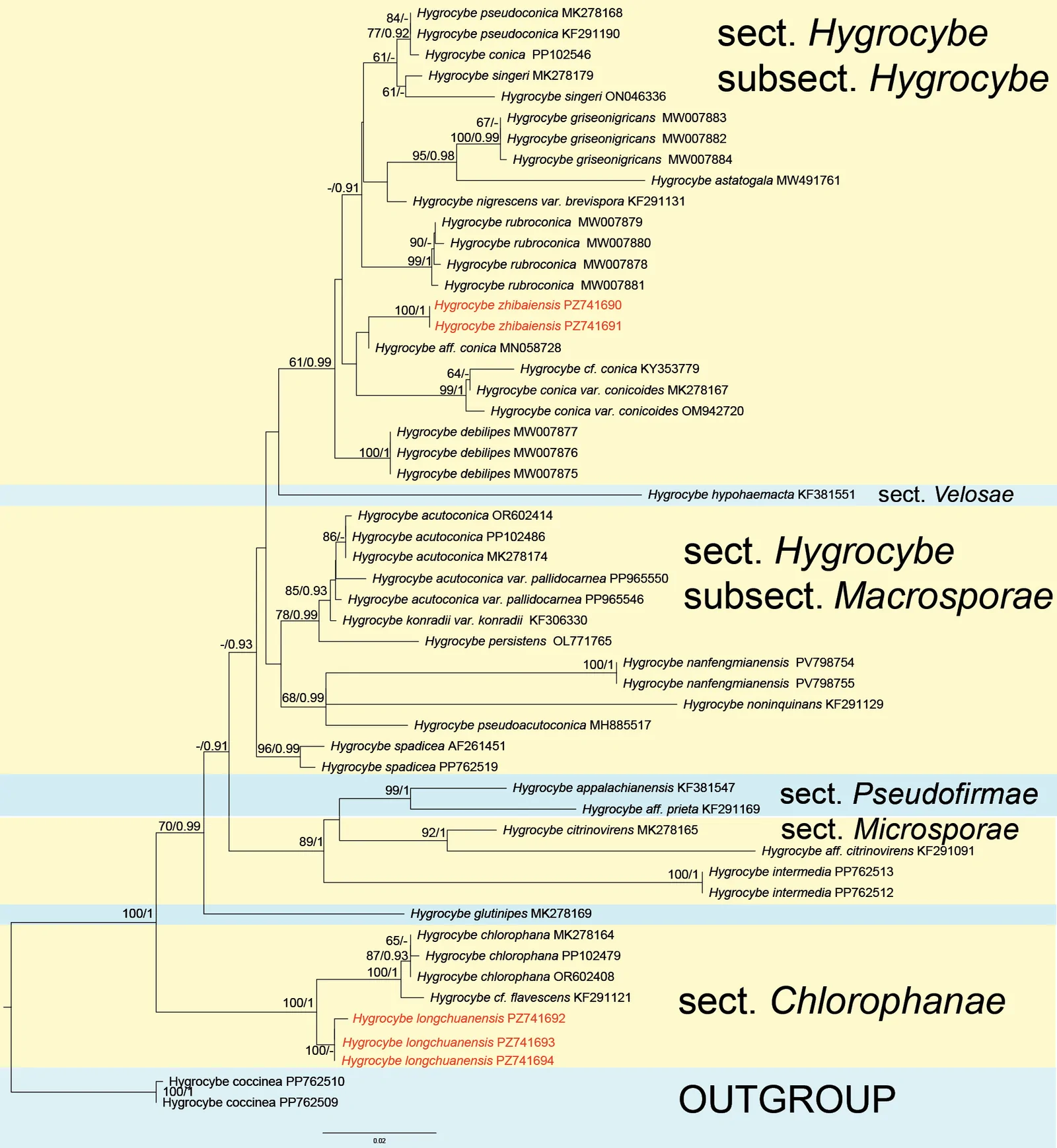
Phylogram generated from maximum likelihood (ML) analysis based on the combined LSU sequence data. Bootstrap support values for maximum likelihood (ML) equal to or greater than 60% and Bayesian posterior probabilities (BYPP) equal to or greater than 0.90 are defined above the nodes as ML/BYPP. New species are indicated in red. The tree is rooted to *Hygrocybe coccinea* (outgroup PP762510, PP762509).

The results of the phylogenetic analysis are shown in Figs 1, 2. Due to the presence of a large number of sequences from unknown and closely related species of *Hygrocybe* in NCBI, we screened the data based on species and sequence reliability, and conducted phylogenetic analyses of ITS and LSU separately. The results showed that in the ITS analysis, *H. zhibaiensis* formed an independent clade with high support (ML = 100%, BI = 1), while the independent clade of *H. longchuanensis* received lower support (ML = 61%, BI < 0.90). In the LSU analysis, the independent clade of *H. zhibaiensis* had low support (ML < 60%, BI < 0.90), whereas *H. longchuanensis* formed an independent clade with high support (ML = 100%, BI = 1).

In this study, we selected 88 sequences for ITS phylogenetic analysis and LSU phylogenetic analysis used 54 sequences. BLAST searches of the holotype ITS sequences in NCBI showed that the most closely related sequence to HFJAU15439 was *Hygrocybe* sp. (HQ022053, percent identity = 94.27%, query cover = 100%), and the most closely related sequence to HFJAU15442 was *Hygrocybe* sp. (OR852467, percent identity = 96.23%, query cover = 100%). BLAST searches of the holotype LSU sequences in NCBI showed that the most closely related sequence to HFJAU15439 was Hygrocybe aff. conica (MN058726, percent identity = 99.07%, query cover = 100%), and the most closely related sequence to HFJAU15442 was Hygrocybe aff. flavescens (MF797678, percent identity = 98.25%, query cover = 100%).

In ITS phylogenetic analysis. The best log-likelihood was -10685.681415. The substitution rate parameters were as follows: AC: 1.244199, AG: 2.673999, AT: 0.865319, CG: 0.860372, CT: 3.647223, GT: 1.000000. The base frequencies were: A: 0.244406, C: 0.234900, G: 0.219600, T: 0.301095; the shape parameter (α) for the Gamma distribution was 0.532270. In LSU phylogenetic analysis. The best log-likelihood was -5596.538. The substitution rate parameters were as follows: AC: 0.97932, AG: 3.28206, AT: 1.34545, CG: 0.74444, CT: 8.54014, GT: 1.00000. The base frequencies were: A: 0.270, C: 0.200, G: 0.287, T: 0.243; the shape parameter (α) for the Gamma distribution was 0.627.

### Taxonomy

#### Hygrocybe zhibaiensis

Taxon classificationFungiAgaricalesHygrophoraceae

D.Y. Xu & D.M. Hu sp. nov.

84214E9D-5BED-596F-8D2C-2DADAF7B147C

Fungal Names: FN 573982

Figs 3, 4

##### Etymology.

“*zhibaiensis*” refers to the type locality, Zhibai Village of Jixi City, Anhui Province, China.

**Figure 3.**
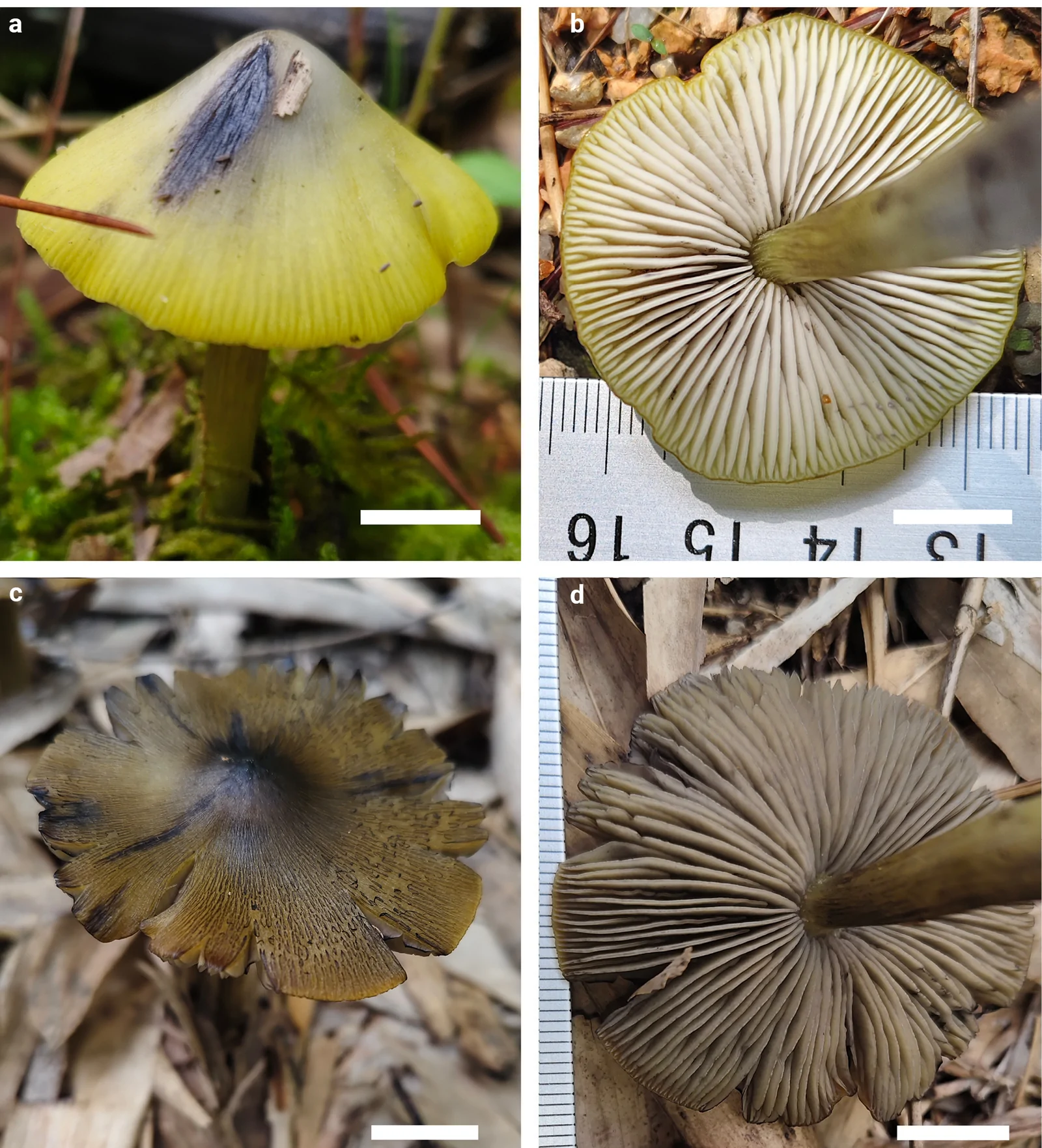
*Hygrocybe zhibaiensis*. **a–d**. Basidiomata (**a, b**. From HFJAU15439 (holotype); **c, d**. From HFJAU15440 (isotype). Scale bars: 10 mm.

**Figure 4.**
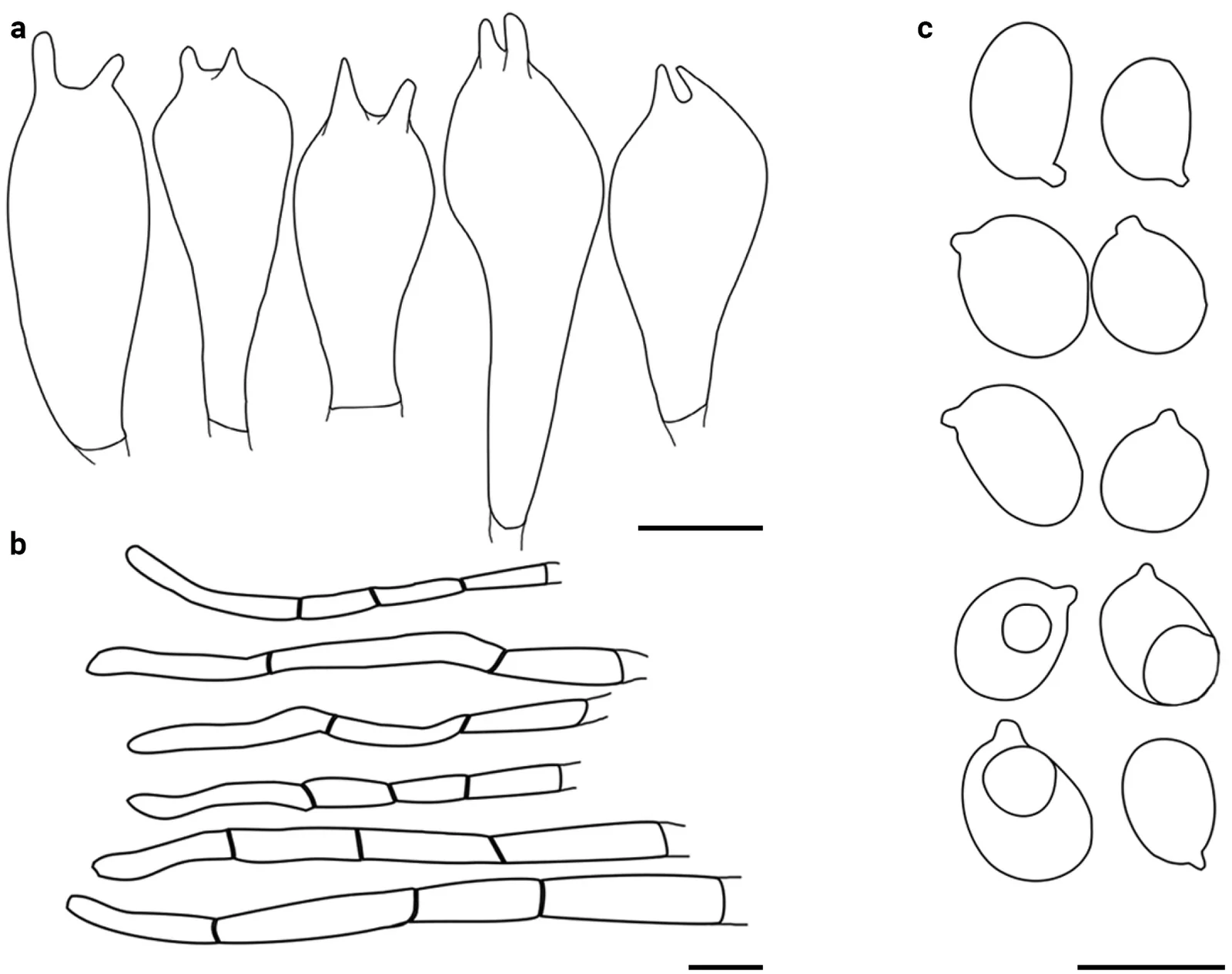
*Hygrocybe zhibaiensis* (HFJAU15439); **a**. Basidia; **b**. Pileipellis; **c**. Basidiospores. Scale bars: 10 μm (**a–c**).

##### Holotype.

Zhibai village Jixi City, Anhui Province, China, 252.01 m alt., 30°5'23"N, 118°43'48"E, 23 Apr. 2025, collected by Dong-Yang Xu, Wen-Tao Guo, HFJAU15439.

##### Diagnosis.

*Hygrocybe zhibaiensis* is characterized by having a greenish pileus; a stipe that is bright yellow at the apex, gradually fading to white downwards; viscid pileus and stipe, and basidiomata that turn black upon bruising or with age; *Hygrocybe conica* differs from *H. zhibaiensis* by reddish orange pileus and yellow stipe; bigger spores and Q value (Basidiospores (9.8)11.5 ± 0.92(13.1) × (6.9)7.5 ± 0.45(8.6) µm, Q = (1.3)1.5 ± 0.12 (1.7)).

##### Description.

Pileus 9–42 mm diam., conical to parabolic when young, becoming conic to campanulate when mature; bright yellowish green (1C8) when fresh, grayish yellow (5D1) to gray (3E1) at center, turning dark red (7D7) or blackish green (2E7) with age or when bruised, with distinct radial striations; surface glutinous when moist, margin acute. Lamellae free, cream yellow (2A2) to white (1A2), crowded (6–7 complete lamellae per cm), with 1–3 lamellulae of unequal length between two complete lamellae; Context thin. Stipe 26–80 × 3–8 mm, with distinct longitudinal striations, bright yellow (3C6) at apex, gradually fading to white (1A1) downwards, turning bluish green (24F6) to dark blue (24E4) when bruised, cylindrical, surface viscid, not easily broken.

Basidiospores (6.1–)6.3–8.4(–8.5) × (7.2–)8.3–10.6(–10.7) μm, Q = (1.0–)1.1–1.6(–1.7), Q_m_ = 1.3, ellipsoid to subglobose, some with a guttule, inamyloid. Basidia (9.9–)10.4–14.3(–15.2) × (25.1–)26.3–32.8(–37.7) μm, clavate, 2 sterigmata, less 1 sterigmata. Lamellar trama regular; hyphae are parallel, with 13.1–27.7 μm wide, no refractive hyphae; oblique septa. Cystidia absent. Pileipellis cutis, hyphae arranged in parallel, 2.8–3.8 μm wide. Stipitipellis hyphae arranged in parallel, 9.2–15.3 μm wide. Clamp connections not observed.

##### Habitat.

Growing gregariously in mixed forests of bamboo and broad-leaved trees.

##### Additional specimens examined.

Longchuan Village, Jixi City, Anhui Province, China, collected by Dong-Yang Xu, Wen-Tao Guo, 211.82 m alt., 30°3'35"N, 118°39'36"E, 23 Apr. 2025, HFJAU15440, isotype. Longchuan Village, Jixi City, Anhui Province, China, collected by Dong-Yang Xu, Wen-Tao Guo, 211.84 m alt., 30°3'35"N, 118°39'36"E, 23 Apr. 2025, HFJAU15441, paratype.

##### Note.

Based on morphological and phylogenetic analyses, *Hygrocybe zhibaiensis* is placed in subsect. *Hygrocybe*. *Hygrocybe zhibaiensis* has a conical to campanulate pileus, free lamellae, and parallel hymenophoral trama hyphae, which are consistent with the characteristics of this subsection. Macroscopically, *H. zhibaiensis* basidiomata are yellowish green when fresh, turning blackish green with age, while *H. conica* basidiomata are reddish orange, turning black with age, and *H. nigrescens* (Quél.) Kühner (1926) basidiomata are orange-yellow and turn black when touched. Microscopically, *H. zhibaiensis* basidiospores and basidia are smaller than *H. conica* (Basidiospores 6.3–8.4 × 8.3–10.6 μm vs 6.5–7.8 × 11.3–13.1 μm; Basidia 10.4–14.3 × 26.3–32.8 μm vs 9.6–12.0 × 41.6–58.9 μm). Compare with *Hygrocybe griseonigricans* C.Q. Wang & T.H. Li, (2020), *H. atrosquamosa*Pegler (1983) and *H. astatogala* (R. Heim) Heinem. (1963), *H. zhibaiensis* has a pileus that is bright yellowish green when fresh, glutinous when moist, and turning dark red or blackish green with age or when bruised; whereas *H. griseonigricans* has a white to pale yellow pileus densely covered with black hairy fibrils and becoming black when bruised or mature, *H. astatogala* has a yellowish-orange to red pileus that appears dull due to black radiating fibrils and is dry, and *H. atrosquamosa* has a pink to orange-red pileus that is yellowish when young and has a smooth surface.

#### Hygrocybe longchuanensis

Taxon classificationFungiAgaricalesHygrophoraceae

D.Y. Xu & D.M. Hu sp. nov.

FF27AFC6-8547-506E-A2E3-D1BEBFBF0341

Fungal Names: FN 573983

Figs 5, 6

##### Etymology.

“*longchuanensis*” refers to the type locality, Longchuan Village of Jixi City, Anhui Province, China.

**Figure 5.**
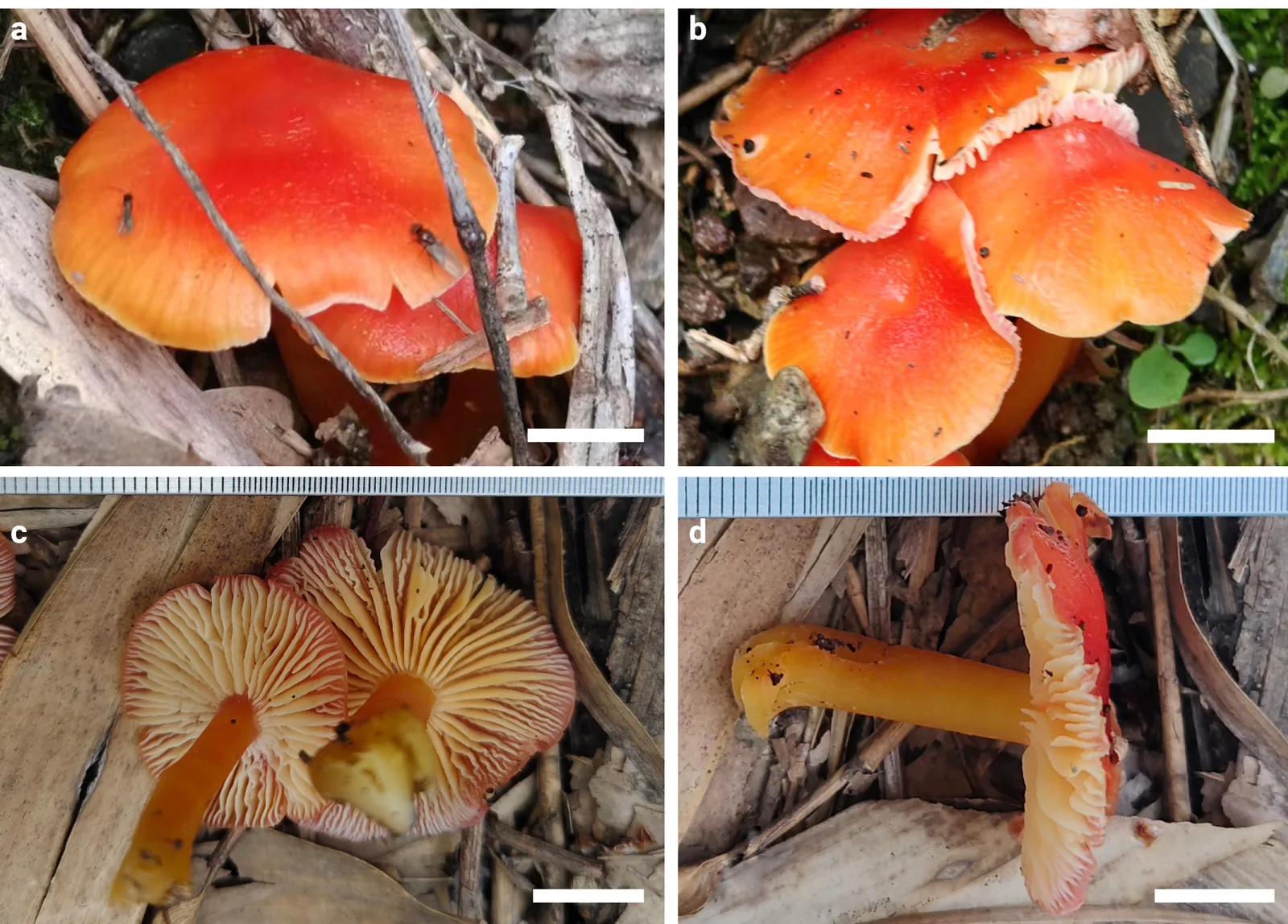
*Hygrocybe longchuanensis*. **a–d**. Basidiomata (**a, c**. From HFJAU15442 (holotype); **b–d**. From HFJAU15445 (isotype)). Scale bars: 10 mm.

**Figure 6.**
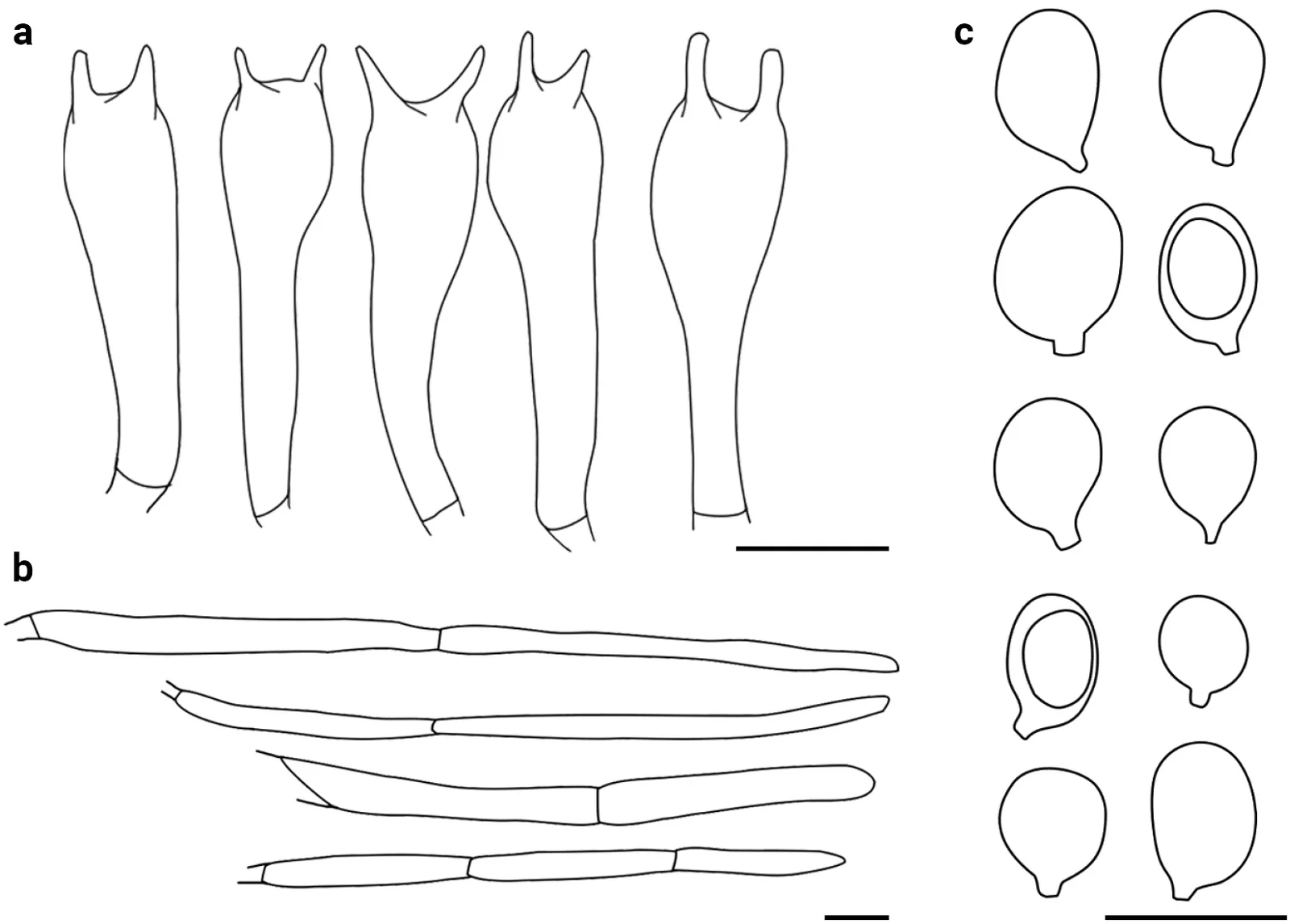
*Hygrocybe longchuanensis* (HFJAU15442). **a**. Basidia; **b**. Pileipellis; **c**. Basidiospores. Scale bars: 10 μm (**a–c**).

##### Holotype.

Anhui Province, Jixi City, China, 219.22 m alt., 30°3'35"N, 118°39'36"E, 23 Apr. 2025, collected by Dong-Yang Xu, Wen-Tao Guo, HFJAU15442.

##### Diagnosis.

*Hygrocybe longchuanensis* is characterized by a bright red pileus at the center with an orange-yellow margin, a viscid surface, and an orange-yellow to yellow stipe, basidiospores ellipsoid to subglobose, no clamp connections. *Hygrocybe longchuanensis* differs from *H. chlorophana* (Fr.) Wünsche (1877) in having smaller basidiomata, a bright red pileus with an orange-yellow margin, whereas *H. chlorophana* has an orange-yellow pileus with a concolorous margin. Furthermore, *Hygrocybe longchuanensis* has ellipsoid to subglobose basidiospores, whereas *H. chlorophana* oblong to ellipsoid basidiospores.

##### Description.

Pileus 11–37 mm in diam., hemispherical, broadly convex to applanate; bright red (8A8) at center, orange-yellow (6A7) at margin; margin with distinct striations; surface glutinous when moist, unchanging when bruised. Lamellae sinuate, cream yellow (4B5) to cream white (4A2), subdistant (6–7 complete lamellae per cm), with 1–4 lamellulae of unequal length between two complete lamellae. Context thin, orange-yellow (4A4) near lamellae, unchanging when exposed to air. Stipe 23–32 × 4–6 mm, cylindrical, orange-yellow (5A6) near apex, gradually fading to yellow (4A6) downwards, brittle, hollow, surface glutinous when moist.

Basidiospores (4.7–)4.9–6.6(–7.0) × (5.2–)6.3–9.0(–9.8) μm, Q = (1.1–)1.2–1.4(–1.5), Q_m_ = 1.3, ellipsoid to subglobose, some with a guttule, inamyloid. Basidia (6.8–)7.1–7.6(–8.7) × (25.3–)26.3–32.0(–35.0) μm, clavate, 2 sterigmata, rarely 1 or 3 sterigmata. lamellar trama regular; hyphae are parallel, with oblique septa, 13.8–21.8 μm wide. Pileipellis is an ixocutis, with parallel hyphae, 7.8–14.4 μm wide. Stipipellis consists of parallel hyphae, 3.1–7.9 μm wide clamp connections not observed.

##### Habitat.

Growing gregariously in mixed forests of bamboo and broad-leaved trees.

##### Additional specimens examined.

Longchuan Village, Jixi City, Anhui Province, China, collected by Dong-Yang Xu, Wen-Tao Guo, 219.22 m alt., 30°3'35"N, 118°39'36"E, 23 Apr. 2025, HFJAU15445, isotype; Jiangxi Ganjiangyuan National Nature Reserve, Shicheng City, Jiangxi Province, China, collected by Dong-Yang Xu, Cheng Guo, Ya-Qing Huang, 532.76 m alt., 26°1'47"N, 116°20'24"E, 21 Apr. 2026, HFJAU15443, paratype; Jiangxi Ganjiangyuan National Nature Reserve, Shicheng City, Jiangxi Province, China, collected by Dong-Yang Xu, Cheng Guo, Ya-Qing Huang, 532.76 m alt., 26°1'47"N, 116°20'24"E, 21 Apr. 2026, HFJAU15444, paratype.

##### Note.

*Hygrocybe longchuanensis* has a bright red pileus, a viscid stipe, and an ixocutis pileipellis, which are consistent with the characteristics of sect. *Chlorophanae*. In phylogenetic analysis, *H. longchuanensis* groups with *H. chlorophana*. Macroscopically, *H. longchuanensis* has a bright red pileus with an orange-yellow margin, which is clearly distinct from *H. chlorophana* (pileus orange-yellow) and *H. flavescens* (Kauffman) Singer (1951) (pileus yellow). Microscopically, the basidiospores of *H. longchuanensis* are larger than those of *H. chlorophana* (4.9–6.6 × 6.3–9.0 μm vs. 4–5 × 7–8.5 μm) and *H. flavescens* (4.9–6.6 × 6.3–9.0 μm vs. 4.5–6 × 7–10 μm), and *H. longchuanensis* spore shape is ellipsoid to subglobose, *H. chlorophana* is ovoid to cylindrical and *H. flavescens* is ellipsoid.

## Discussion

Morphologically, *Hygrocybe zhibaiensis* conforms to the characteristics of sect. *Hygrocybe* subsect. *Hygrocybe*, and *H. longchuanensis* conforms to the characteristics of sect. *Chlorophanae* (Lodge et al. 2014). In the ITS phylogenetic analysis, *H. zhibaiensis* forms a strongly supported independent clade within subsect. *Hygrocybe*; in the LSU phylogenetic analysis, *H. longchuanensis* forms a strongly supported independent clade within sect. *Chlorophanae*. Both species exhibit distinct characteristics that clearly differ from other species in their respective sections; therefore, we place *H. zhibaiensis* in subsect. *Hygrocybe* and *H. longchuanensis* in sect. *Chlorophanae*.

*Hygrocybe* exhibits considerable regional variation, and most specimens are commonly treated as European species (Chen and Li 2013). Additionally, some species closely resemble *Hygrophorus* in morphology, which has led to numerous misidentifications (Wang et al. 2023). The *Hygrocybe* is distributed throughout China (Chen and Li 2013; Wang et al. 2019). In recent years, dozens of articles reporting the discovery of new *Hygrocybe* species have been published in China (Wang et al. 2013, 2015, 2019, 2020, 2021; Guo et al. 2025; He et al. 2026; Li and Yang 2026), which fully demonstrates that the diversity of *Hygrocybe* in China is remarkably rich. In this study, the collection localities of *H. longchuanensis* are more than 600 km apart, suggesting that this is a widely distributed species in southeastern China. This may indicate that many widely distributed but yet undiscovered *Hygrocybe* species still exist in southeastern China. In addition, the two specimens of *H. zhibaiensis* were collected on opposite sides of a mountain range, with a straight-line distance of more than 10 km between them. This suggests that it is a widely distributed species in Jixi City. Although no specimens were collected in the Qingliangfeng National Nature Reserve, it can be inferred that the species also occurs there. This may suggest that the diversity of *Hygrocybe* in southwestern China shows little variation in horizontal distribution. To test this hypothesis, however, more time and effort are needed for further in-depth study.

## Supplementary Material

XML Treatment for Hygrocybe zhibaiensis

XML Treatment for Hygrocybe longchuanensis

## Additional information

### Artificial Intelligence (AI) use

The authors accept full responsibility for the content of the manuscript, including the disclosure of any use of AI.

No AI tools were used in the preparation of this manuscript.

### Author ORCIDs

D.-Y. Xu https://orcid.org/0009-0006-6122-7833

X.-C. Hu https://orcid.org/0009-0000-3846-7640

J. Liu https://orcid.org/0009-0005-5237-6629

J.-P. Zhou https://orcid.org/0009-0003-2511-7686

M.-H. Chen https://orcid.org/0009-0006-3497-3312

Z.-J. Zhai https://orcid.org/0009-0008-7562-9707

J.-E. Huang https://orcid.org/0000-0003-3627-3193

C.-W. Liu https://orcid.org/0009-0002-2247-1180

H.-Y. Song https://orcid.org/0009-0001-4140-5720

D.-M. Hu https://orcid.org/0000-0002-4750-2871

### Data availability

All of the data that support the findings of this study are available in the main text.
